# Pediatric Pseudoaneurysm of the Iliac Artery Following a Mini‐Laparoscopic Surgery: A Case Report

**DOI:** 10.1155/crpe/3532373

**Published:** 2025-12-17

**Authors:** Dianzhu Ding, Jikuan Li, Yanbo An, Xiaoming Shi

**Affiliations:** ^1^ Department of Vascular Surgery, Hebei General Hospital, Shijiazhuang, Hebei, China, hebmu.edu.cn

**Keywords:** iliac artery, laparoscopic, pediatric, pseudoaneurysm

## Abstract

Pediatric pseudoaneurysm of the iliac artery is extremely uncommon. In many cases, pseudoaneurysms may be caused by trauma, infection, or arterial catheterization. We report a rare case of an iliac artery pseudoaneurysm in a 3‐year‐old boy following a mini‐laparoscopic surgery for the treatment of hydrocele. Which is the first reported case of pediatric laparoscopic‐related iliac artery injury; moreover, he was successfully cured by conservative treatment. The patient presented with mild pain in the abdomen. The hemodynamic was stable. Ultrasound and contrast‐enhanced computed tomography confirmed an iliac artery pseudoaneurysm with partial thrombus. With daily ultrasound monitoring and intermittent compression, the child was successfully treated without any surgery. This case revealed iliac artery injury might happen during mini‐laparoscopic surgery. If symptoms were mild or emergency operation could be performed at any time, daily ultrasound monitoring and intermittent compression methods could be carried out on a pediatric iliac artery pseudoaneurysm patient.

## 1. Introduction

With recent innovations in laparoscopic instrumentation and advances in surgical techniques, minimally invasive surgery—particularly mini‐laparoscopy—has gained increasing popularity in pediatric surgery. This approach is now commonly employed in the management of appendicitis, hernia repairs, and urological conditions in children, owing to its benefits such as reduced postoperative pain, shorter hospital stays, and improved cosmetic outcomes. However, despite its overall safety profile, laparoscopic surgery is not without risk. Vascular injuries, though rare, represent some of the most severe complications associated with these procedures.

In this report, we present the case of a 3‐year‐old child who developed a right common iliac artery pseudoaneurysm following iatrogenic injury during laparoscopic surgery. Successful occlusion was achieved using ultrasound‐guided compression therapy. To our knowledge, this is the first reported instance of complete regression of an iatrogenic iliac artery pseudoaneurysm in a preschool‐aged child managed with this minimally invasive technique.

## 2. Case Report

**Figure 1:**
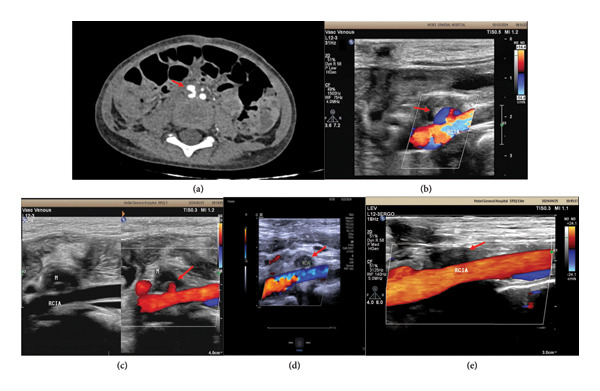
(a) February 9, 2024. Abdominal enhanced CT revealed rupture of the anterior wall of the right common iliac artery with pseudoaneurysm formation. (b) February 13, 2024 (Day 4), color Doppler ultrasound of the iliac artery demonstrated stable pseudoaneurysm dimensions, partial intraluminal thrombosis, and reduced flow cavity volume. (c) February 27, 2024 (Day 18), follow‐up iliac artery color Doppler ultrasound demonstrated significant reduction in the pseudoaneurysm cavity size. (d) March 22, 2024 (Day 42), follow‐up iliac artery color Doppler ultrasound demonstrated complete thrombosis of the pseudoaneurysm. (e) April 25, 2024 (Day 72), follow‐up imaging revealed complete resolution of the iliac artery pseudoaneurysm.

A 3‐year‐old male underwent laparoscopic hydrocelectomy at an external institution. Intraoperatively, a right retroperitoneal hematoma was identified without active bleeding, prompting procedure termination. On postoperative Day 3, the child developed acute abdominal pain with crying. Ultrasound revealed hematoma expansion accompanied by tachycardia and hypotension, necessitating emergent blood transfusion and exploratory laparotomy. Eleven days after the first operation, recurrent abdominal pain prompted repeat ultrasonography, demonstrating a right common iliac artery pseudoaneurysm. The patient was subsequently transferred to our center. Emergency contrast‐enhanced abdominal CT confirmed the diagnosis (Figure 1(a)).

Multidisciplinary consensus generated three therapeutic options: (1) endovascular therapy: balloon occlusion of the right common iliac artery with microcatheter‐directed thrombin injection; (2) open surgical repair: pseudoaneurysm resection with arterial wall reconstruction; (3) conservative management: ultrasound‐guided transabdominal compression with serial monitoring.

Following family consultation, conservative treatment was elected. This decision was considered as follows: Paroxysmal abdominal pain temporally associated with feeding (suggestive of functional pain or adhesive disease), localized pseudoaneurysm morphology without rupture signs on CT, and stable hemoglobin levels. The implemented protocol included daily Doppler ultrasound surveillance, ultrasound‐guided abdominal compression (30 min/day), activity restriction, and preoperative preparation for emergent intervention.

The details of ultrasound‐guided compression therapy for pseudoaneurysms are as follows: (1) It is necessary to establish a good relationship with young children, toys or snacks can be brought to the child before each treatment, and used to build trust and distract the child during treatment; (2) ultrasound accurately locates the body surface projection of the abdominal wall before compression and gently pushes the intestine to achieve the purpose of directly pressing the iliac artery rupture through the abdominal wall; (3) at the same time of pressing, the assistant touches the ipsilateral femoral artery pulsation to avoid the ipsilateral lower extremity artery being completely blocked; (4) this method is suitable for children with thin body.

By Day 4, surveillance demonstrated: stable pseudoaneurysm dimensions, partial intraluminal thrombosis, reduced flow cavity volume (Figures 1(b)), and complete resolution of abdominal pain. Compression therapy was discontinued with ambulation permitted (strenuous activity prohibited). Subsequent evaluations showed the following: Day 18: significant cavity reduction (Figures 1(c)); Day 42: absent flow signals with further size regression (Figures 1(d)); Day 72: complete ultrasonographic resolution with normal iliac arterial flow (Figures 1(e)). The iliac artery pseudoaneurysm was successfully treated and resolved.

## 3. Discussion

Aneurysms are defined as a permanent localized dilatation of an artery having at least 50% increases in diameter compared with the expected normal diameter [1]. Pseudoaneurysms do not involve the entire arterial wall. Therefore, pseudoaneurysms are more likely to rupture than true aneurysms. Iatrogenic injury is one of the common causes of pseudoaneurysm. According to case reports and reviews, artery injuries related to laparoscopic surgery include inferior epigastric artery, cystic artery, uterine artery, and abdominal aorta [2–5]. To our knowledge, this case is the first report of iliac artery pseudoaneurysm caused by laparoscopic surgery. Interventional embolization or surgical repair represents the standard management for iliac artery pseudoaneurysms. Case report and literature review showed the success of interventional embolization [6, 7]. However a review of surgical treatment of iatrogenic iliofemoral artery injury in the pediatric population revealed failure and death of surgical treatment [8]. And in this pediatric case with two recent consecutive intra‐abdominal surgery, repeat surgical posed significant risks of adhesion‐related complications, such as small intestine rupture, adhesive intestinal obstruction, and high risk of failure to fix the iliac artery due to the adhesion around the iliac artery and edema. Endovascular approaches also presented considerable challenges, with non‐negligible failure rates in such complex scenarios. The application of covered stents is limited in pediatric patients due to anatomical constraints, and embolization therapy is associated with the potential for distal limb embolism. Concomitant progressive resolution of abdominal pain further supported conservative management as a viable therapeutic alternative. With the overall consideration, conservative management was taken. It should be emphasized that the emergency surgery for the child must be ready and can be performed at any time.

While laparoscopic procedures demonstrate low arterial injury rates, iliac artery pseudoaneurysms remain a critical albeit rare complication. Specific technical adaptations are warranted: (a) preoperative Doppler mapping of aorta artery and iliac bifurcations; (b) trocar insertion angles > 60° from major vessels. These methods may reduce the incidence of arterial injury. Once a retroperitoneal hematoma is identified during surgery, or if bleeding from any site is suspected, meticulous and precise hemostasis must be obtained. In cases of iliac artery pseudoaneurysm, conservative management may be a reasonable option provided the patient is hemodynamically stable, reports no significant abdominal or lumbar pain, and imaging (CT or ultrasound) demonstrates a contained lesion without signs of active extravasation or surrounding inflammation—particularly when both surgical and interventional treatments are deemed to carry high risks.

## 4. Conclusion

This case aims to heighten vigilance for such injuries and mitigate iatrogenic risks in pediatric minimally invasive surgery. That in carefully selected, hemodynamically stable pediatric patients, a conservative approach with close monitoring can be a safe and effective alternative to more invasive treatments for iatrogenic pseudoaneurysms.

## Ethics Statement

This case report was conducted in accordance with the Declaration of Helsinki and was approved by the Institutional Review Board/Ethics Committee of Hebei General Hospital (Approval No.: 2025‐LW‐0157). All the patients allowed personal data processing and informed consent was obtained from all individual participants included in the study.

## Conflicts of Interest

The authors declare no conflicts of interest.

## Funding

This research did not receive any specific grant from funding agencies in the public, commercial, or not‐for‐profit sectors.

## Data Availability

The data that support the findings of this study are available from the corresponding author upon reasonable request.
